# Global Wild Annual *Lens* Collection: A Potential Resource for Lentil Genetic Base Broadening and Yield Enhancement

**DOI:** 10.1371/journal.pone.0107781

**Published:** 2014-09-25

**Authors:** Mohar Singh, Ishwari Singh Bisht, Sandeep Kumar, Manoranjan Dutta, Kailash Chander Bansal, Moreshwar Karale, Ashutosh Sarker, Ahmad Amri, Shiv Kumar, Swapan Kumar Datta

**Affiliations:** 1 National Bureau of Plant Genetic Resources, Pusa, New Delhi, India; 2 International Centre for Agricultural Research in Dry Areas, South Asia and China Regional Programme, NASC Complex, DPS Marg, Pusa, New Delhi, India; 3 International Centre for Agricultural Research in Dry Areas, Tunisia, Tunis; 4 International Centre for Agricultural Research in Dry Areas, Rabat, Morocco; 5 Division of Crop Science, Indian Council of Agricultural Research, Krishi Bhawan, New Delhi, India; China Agricultrual University, China

## Abstract

Crop wild relatives (CWRs) are invaluable gene sources for various traits of interest, yet these potential resources are themselves increasingly threatened by the impact of climate change as well as other anthropogenic and socio-economic factors. The prime goal of our research was to cover all aspects of wild *Lens* genetic resource management like species characterization, agro-morphological evaluation, diversity assessment, and development of representative sets for its enhanced utilization in lentil base broadening and yield improvement initiatives. We characterized and evaluated extensively, the global wild annual *Lens* taxa, originating from twenty seven counties under two agro-climatic conditions of India consecutively for three cropping seasons. Results on various qualitative and quantitative characters including two foliar diseases showed wide variations for almost all yield attributing traits including multiple disease resistance in the wild species, *L. nigricans* and *L. ervoides* accessions. The core set developed from the entire *Lens* taxa had maximum representation from Turkey and Syria, indicating rich diversity in accessions originating from these regions. Diversity analysis also indicated wide geographical variations across genepool as was reflected in the core set. Potential use of core set, as an initial starting material, for genetic base broadening of cultivated lentil was also suggested.

## Introduction

Crop wild relatives (CWRs) are the reservoir of useful genes and alleles that can be used in breeding new and better adapted varieties resistant to biotic and abiotic stresses, and more importantly to adapt to adverse effect of climate change. [1], [2]. Despite their potential value, many CWRs are, however, not adequately collected and conserved in gene banks across the world. Increasing threats to natural habitats and farming systems makes it imperative to collect, conserve and characterize CWRs in order to make them available for use in mitigating the impact of major biotic and abiotic stresses caused by climate change [1] and other factors. The genus *Lens* Miller is part of the family *Fabaceae* (*Leguminosae*), and is placed in either subfamily *Faboideae* tribe *Fabeae* [3] or in subfamily *Papilionaceae* tribe *Vicieae* [4]. Lentil is an annual self-pollinating true diploid (2n = 2x = 14) species with an estimated genome size of 4063 Mbp/C [5]. The cultivated lentil (*Lens culinaris* Medikus ssp. *culinaris*) encompasses two groups on the basis of agro-morphological traits, the small-seeded (*microsperma*), large-seeded (*macrosperma*) and *L. culinaris* ssp. *orientalis* (Boiss.) Ponert, is considered its immediate wild progenitor. The other sub-species and species are *L. culinaris* ssp. *tomentosus* (Ladiz.), *L. culinaris* ssp. *odemensis* (Ladz.), *L. ervoides* (Brign.), *L. nigricans* (Bieb.) and *L. lamottei* Czefr. [6]. Globally lentil productivity has increased from an average yield of 560 kg ha^−1^ in 1961–63 to 950 kg ha^−1^ during 2010–2011. Despite this increase, the current yields are much lower as compared to many other grain legume species, because of the limited yield potential of modern lentil cultivars. Major yield limiting factors include, poor seedling vigour, slow leaf area development, high rate of flower drop, low pod setting, poor dry matter, low harvest index, lack of lodging resistance, and exposure to several biotic and abiotic stresses [7]. Further, lentil breeding programme in the past has been primarily depended on landraces of cultivated lentil. Recently developed lentil varieties, however, have superiority over traditional cultivars in terms of their yield potential and disease resistance. For the development of these modern varieties, a small number of improved local landraces have been utilized mainly through pure-line selection following hybridization between lines adapted to specific environmental conditions. Substantial improvement in the lentil productivity could not be achieved due to the loss of invaluable alleles for higher productivity including resistance/tolerance to major biotic and abiotic stresses and low extent of genetic variation in lentil cultivars [8]–[11]. Wild relatives of lentil are good sources of disease resistance as well as many useful agronomic characters like, high number of pods plant^−1^, high number of pods cluster^−1^, high number of branches plant^−1^, and short internodes [12], [13]. Furthermore, substantial genetic diversity is currently not available in *ex-situ* conserved collections. The treasure of lentil genetic resource is held up in populations poorly or incompletely sampled or completely unsampled wild *Lens* taxa. The gap not only limits its use, it also renders precious genetic diversity inaccessible and vulnerable to extinction [14]. Maxted et al. (2012a) [15] suggested a comprehensive strategy for gap analysis on CWRs including lentil for germplasm conservation and how existing georeferenced passport data associated with the collections of *Lens* species can be utilized in identifying gaps in the current *ex-situ* holdings and develop reliable *in-situ* conservation strategy.

The efficient utilization of desired diversity of crop wild relatives will be the solution to achieve substantial gains in productivity of cultivated gene pool. Maintaining and evaluating a large set of accessions of wild *Lens* species is often difficult due to various reasons like inadequate seed germination, high pod shattering and photoperiod sensitivity and certain wild *Lens* species do not flower under normal winter cropping season. These photoperiod sensitive accessions would require artificially lengthened days in the greenhouse or growing them under long days in summer season.

Establishing a core collection of the existing holding is a favoured approach for efficient utilization of novel variations in wild gene pool with a manageable number. The core collection is a limited set of accessions representing, with minimum repetitiveness, the genetic diversity of a crop and its wild relatives [16]–[19]. Genebank curators have the responsibility for conservation, regeneration, safety duplication, documentation, characterization and evaluation as well as facilitating utilization of the genetic resources to the breeder for crop improvement. The core collection, being smaller in size in comparison to the whole collection, can be used very effectively as a starting point for research involving screening of the germplasm collection for sources of desirable traits. Since, the inception of the idea of core collections, many approaches for selecting core collections have been proposed and used e.g. M-Strat [20], Genetic Distance Sampling [21], PowerCore [22] and Core Hunter [23]. Similarly, core has been developed using several kind of data ranging from genealogical data in the Czech spring wheat [24], agronomic data in groundnut [25], [26] and molecular data or integration of data, in bread wheat [27] and in rice [28], [29]. The core collection of wild *Lens* gene pool could serve as a working collection for the users, which then needs to be extensively evaluated and used in lentil improvement. Focused Identification of Germplasm Strategy (FIGS approach), based on co-evolution between the plant and the environment conditions, uses trait and environmental data (climate data and phenological data) to develop *a priori* information based on the quantification of the trait-environment relationship. This relationship when detected can be used to develop subsets of accessions with a high probability of containing the sought after traits. This approach was used efficiently in wheat, barley and faba bean to select novel sources of resistance and tolerance to diseases, insect, drought and salinity [30], [31].

In view of the above, the objectives of the present study were (1) to systematically characterize and evaluate global wild annual *Lens* collection for different morphological and agronomic traits including major foliar biotic stresses, (2) to develop core set of wild *Lens* taxa for enhanced utilization in lentil genetic base broadening and yield enhancement, (3) to assess the diversity available in global wild annual *Lens* collection, and compare it with that of core set and (4) to analyse the geographical distribution of wild *Lens* taxa representing maximum diversity, and suggest potential areas for future collecting and conserving diversity *in-situ* on-farm.

## Materials and Methods

### Plant material

A total of 405 diverse global wild annual *Lens* accessions comprising 4 of cultivated *L. culinaris* ssp. *culinaris*, 171 of *L. culinaris* ssp. *orientalis*, 42 of *L. culinaris* ssp. *odemensis*, 20 of *L. culinaris* ssp. *tomentosus*, 35 of *L. nigricans*, 124 of *L. ervoides* and 9 of *L. lamottei* (Table 1) were studied encompassing various aspects of genetic resource management. These include species characterization and agro-morphological evaluation, development of core sets for its enhanced utilization in lentil genetic base broadening, diversity assessment, and geographical distribution of *Lens* taxa representing maximum diversity. The wild *Lens* accessions were obtained from the Genebank of the International Centre for Agricultural Research in the Dry Areas (ICARDA) Aleppo, Syria. The original identity of accessions in the present study was the same as established by the ICARDA Genetic Resources Unit.

**Table 1:** List of wild annual *Lens* accessions introduced from ICARDA, Aleppo, Syria.

S. No.	Species	Accessions	Origin
1	*Lens culinaris* ssp. *culinaris*	04	India, Israel
2	*Lens culinaris* ssp. *orientalis*	171	Turkey, Iran, Cyprus, Israel, Syria, Jordan, Uzbekistan, Tajikistan, Turkmenistan, Lebanon, Armenia, Azerbaijan, Russia, Czech Republic
3	*Lens culinaris* ssp. *odemensis*	42	Syria, Palestine, Turkey, Israel
4	*Lens culinaris* ssp. *tomentosus*	20	Turkey, Syria
5	*Lens nigricans*	35	Syria, Italy, France, Spain, Croatia, Bahrain, Turkey, Ukraine, Montenegro, Croatia
6	*Lens ervoides*	124	Ukraine, Turkey, Croatia, Italy, Former Soviet Union, Bahrain, Israel, Palestinian Territories, Syrian, Jordan, Tajikistan, Lebanon, Former Yugoslavia, Montenegro, Armenia, Azerbaijan
7	*Lens lamottei*	09	Spain, Turkey, France

### Species characterization and agronomic evaluation

All 405 global annual *Lens* accessions including four commercial lentil cultivars were grown at two agro-ecological regions of India, the National Bureau of Plant Genetic Resources (NBPGR), New Delhi, India during the winter (*rabi*) season of 2011–2012, 2012–2013 and 2013–2014 for characterization and agro-morphological evaluation and the summer Himalayan nursery (*summer* 2012 and 2013) at Himachal Pradesh Agricultural University (HPAU), Research Station (RS), Sangla. The permission to conduct experiments at Sangla was granted by worthy Vice-Chancellor of the University. The location of NBPGR lies in the northern plain between 28^0^ 35′ N latitude, 70^0^ 18′ E longitude, 226 m asl altitude, whereas the HPAU, RS, Sangla lies between latitudes 31^0^ 55′ and 32^0^ 20′ N, and longitudes 77^0^ 00′ and 79^0^ 50′ E, in the northwestern Himalayas. Each accession was sown by hand in three replications of 3 m long rows spaced 30 cm apart. The soil type was sandy loam at both the locations. All these annual *Lens* species were characterized for qualitative morphological traits viz; seedling stem pigmentation, leaf pubescence, leaflet size, tendril length, pod shedding, pod dehiscence, flower colour, ground colour of testa, pattern of testa and cotyledon colour using standard lentil descriptors [32]. Observations were also recorded on ten plants accession^−1^ in each replication for ten quantitative morphological traits viz; days to flowering, days to maturity, plant height (cm), number of branches plant^−1^, number of pods plant^−1^, number of seeds pod^−1^, number of seeds plant^−1^, 100-seed weight (g), seed yield plant^−1^ (g) and biological yield plant^−1^(g). The data were subjected to statistical analysis using MS Ofiice Excel and SAS software.

### Screening against foliar biotic stresses

#### Rust (Uromyces fabae (Grev.) Fuckel)

Reaction to *Uromyces fabae* was conducted at the Himachal Pradesh Agricultural University Research and Extension Centre (HPAUREC), Dhaulakuan (30^0^ 4′ N latitude, 77^0^ 5′ longitudes, 468 m asl) located in the foot hills of northwestern Himalayas. All the accessions were grown in 25 cm diameter plastic pots in soil and farmyard manure mixture (10∶1). Each accession had two replications. Pots with plants at the vegetative stage were transferred in the field and were artificially inoculated by frequently spraying with ascosporic suspension (1×10^6^ spores/ml) using the isolate of *Uromyces fabae.* The data were recorded on terminal disease reaction at vegetative and reproductive plant stages using 1–9 rating scale as suggested by [33]. Based on disease score, the wild accessions were categorized for their reaction to rust infection as: 1 = asymptomic (Free); 3 = resistant (R); 5 = moderately resistant (MR); 7 = susceptible (S); and 9 = highly susceptible (HS).

#### Powdery mildew (*Erysiphe polygoni DC*.)

Powdery mildew is an economically important foliar disease of lentil especially in those areas where warm and humid weather persists. For the screening of wild *Lens* species, all accessions were grown in 25 cm diameter plastic pots in soil and farmyard manure mixture (10∶1), and planted pots were kept and maintained in the glasshouse. The disease appeared at flower initiation stage, which was further allowed to develop. The pots with heavily infected plants were shifted near to test entries grown in the glass house. The heavily infected plants were shaken well over the test entries in the evening time to disperse conidia, which helps in spreading the disease on the test entries as suggested by [34]. Data on disease incidence were recorded using 1–9 rating scale at vegetative and reproductive stages as: 1 = asymptomic (Free); 3 = resistant (R); 5 = moderately resistant (MR); 7 = susceptible (S); and 9 = highly susceptible (HS).

### Development of core set

The entire set of *Lens* species was used for developing a core collection using “PowerCore” (http://genebank.rda.go.kr/powercore/) software developed at the Rural Development Administration (RDA), South Korea [22]. Separate sets were developed using both quantitative and qualitative data in combination as well as in isolation. “PowerCore” creates subsets representing all alleles or observation classes, with the least allelic redundancy, and ensures a highly reproducible list of entries. The Shannon Diversity Index [35] was used to compare the representativeness of the entire holding and the accessions selected as core entries.

### Diversity analysis

Diversity analysis was conducted using both qualitative and quantitative phenotypic data of complete set of 405 accessions as well as of the core set developed from it. Genetic relationships among accessions were determined by means of Dice’s coefficient [36] in case of qualitative data. Phenotypic relationships based on quantitative data were determined from the Euclidean distances. Trees were constructed using hierarchical clustering method by means of Jaccard’s coefficient with UPGMA mode with the DARwin 5.0 software [37], [38].

## Results

All 405 accessions of global annual *Lens* taxa including four commercial cultivars were characterized and evaluated for morphological and agronomic traits as well as for two major fungal diseases under two agro-climatic regions in the north-western Indian conditions.

### Species characterization using qualitative traits

The frequency distribution of seven annual *Lens* species showed a wide range of intraspecific variation for majority of the important morphological plant characteristics (Table 2). Seedling stem pigmentation exhibited variations in all the *Lens* species except, *L. culinaris* ssp. *culinaris*, where it was absent. Likewise, leaf pubescence, leaflet size and tendril length also revealed remarkable variation in all seven annual *Lens* taxa. There was substantial variation in pod shedding and dehiscence for majority of the *Lens* species except, *L. culinaris* ssp. *culinaris*. Flower colour was white and purple in majority of the *Lens* species like, *L. culinaris* ssp. *culinaris*, *L. culinaris* ssp. *orientalis*, *L. nigricans* and *L. lamottei* and in *L. culinaris* ssp. *odemensis*, *L. culinaris* ssp. *tomentosus*, and in *L. ervoides*, it was purple only. The ground colour of testa was mostly grey and brown in all the *Lens* species except, *L. culinaris* ssp. *culinaris*, where it was all brown. Substantial variations were also found in the pattern of testa for all the annual *Lens* species. All the annual *Lens* species had yellow and orange cotyledon colour except, *L. culinaris* ssp. *tomentosus,* where it was all orange (Table 2).

**Table 2:** Frequency distribution of qualitative plant characteristics in different annual *Lens* species.

Character	Class (descriptor state)	Frequency						
		*L. culinaris* ssp. *culinaris*	*L. culinaris* ssp. *orientalis*	*L. culinaris* ssp. *odemensis*	*L. culinaris* ssp. *tomentosus*	*L. nigricans*	*L. ervoides*	*L. lamottei*
**Seedling stem pigmentation**	0: Absent	1.00	0.09	0.03	0.11	0.54	0.15	0.43
	1: Present	0.00	0.91	0.97	0.89	0.46	0.85	0.57
**Leaf pubescence**	0: Absent	0.00	0.03	0.00	0.00	0.14	0.30	0.29
	3: Slight	0.75	0.50	0.35	0.21	0.64	0.66	0.71
	7: Dense	0.25	0.47	0.65	0.79	0.22	0.04	0.00
**Leaflet size**	3: Small	0.00	0.53	0.30	0.16	0.82	0.37	0.29
	5: Medium	0.50	0.46	0.68	0.79	0.14	0.49	0.71
	7: Large	0.50	0.01	0.02	0.05	0.04	0.14	0.00
**Tendril length**	1: Rudimentary	0.17	0.29	0.14	0.11	0.25	0.33	0.71
	2: Prominent	0.83	0.71	0.86	0.89	0.75	0.67	0.29
**Pod shedding**	0: None	1.00	0.00	0.00	0.00	0.00	0.00	0.00
	3: Low	0.00	0.11	0.20	0.00	0.23	0.11	0.50
	5: Medium	0.00	0.28	0.60	0.36	0.54	0.26	0.33
	7: High	0.00	0.61	0.20	0.64	0.23	0.63	0.17
**Pod dehiscence**	0: None	1.00	0.00	0.00	0.00	0.00	0.00	0.00
	3: Low	0.00	0.00	0.20	0.00	0.18	0.11	0.33
	5: Medium	0.00	0.90	0.80	0.64	0.64	0.56	0.50
	7: High	0.00	0.10	0.00	0.36	0.18	0.33	0.17
**Flower colour**	1: White	0.17	0.09	0.00	0.00	0.22	0.00	0.29
	2: Yellow	0.00	0.00	0.00	0.00	0.00	0.00	0.00
	3: Red	0.00	0.00	0.00	0.00	0.00	0.00	0.00
	4: Purple	0.83	0.91	1.00	1.00	0.78	1.00	0.71
**Ground colour of testa**	1: Green	0.00	0.00	0.00	0.00	0.00	0.00	0.00
	2: Grey	0.00	0.07	0.35	0.06	0.37	0.26	0.57
	3: Brown	1.00	0.93	0.65	0.94	0.63	0.74	0.43
	4: Black	0.00	0.00	0.00	0.00	0.00	0.00	0.00
	5: Pink	0.00	0.00	0.00	0.00	0.00	0.00	0.00
**Pattern of testa**	0: Absent	0.50	0.01	0.00	0.06	0.00	0.04	0.00
	1: Dotted	0.50	0.17	0.05	0.00	0.11	0.08	0.00
	2: Spotted	0.00	0.12	0.11	0.06	0.15	0.08	0.14
	3: Marbled	0.00	0.58	0.16	0.76	0.59	0.74	0.57
	4: Complex	0.00	0.12	0.68	0.12	0.15	0.06	0.29
**Cotyledon colour**	1: Yellow	0.25	0.04	0.78	0.00	0.19	0.10	0.71
	2: Orange	0.75	0.96	0.22	1.00	0.81	0.90	0.29
	3: Olive-green	0.00	0.00	0.00	0.00	0.00	0.00	0.00

### Agronomic evaluation using quantitative traits

All the annual *Lens* accessions were evaluated for various agro-morphological quantitative traits. The results revealed significant variations (p = 0.05) as was evident from the analysis of variance as also the range, mean and coefficient of variations for all traits studied (Table 3 and 4). In general, the coefficient of variation ranged from low (days to flowering and maturity) to high (number of branches^−1^, number of pod plant^−1^ and number of seeds pod^−1^) for important agro-morphological traits in all the cropping seasons. However, the mean performance for days to flowering and maturity was lowest in *L. culinaris* ssp. *culinaris*, followed by *L. culinaris* ssp. *orientalis*. Likewise, maximum plant height was recorded in *L. lamottei and L. nigricans* and lowest in *L. culinaris* ssp.*culinaris*. The mean number of branches plant^−1^ was highest in *L. ervoides* followed by *L. culinaris* ssp. *tomentosus, L. culinaris* ssp. *odemensis*, *L. lamottei* and *L. culinaris* ssp. *orientalis*, and lowest in *L. culinaris* ssp. *culinaris*. There was also substantial variation in the range for number of pods plant^−1^ with maximum number of pods plant^−1^ recorded in *L. ervoides* followed by *L. culinaris* ssp. *culinaris*, *L. culinaris* ssp. *odemensis*, *L. culinaris* ssp. *tomentosus* and *L. culinaris* ssp. *orientalis*. The number of seeds pod^−1^ was >1 in all the annual *Lens* species. As expected, cultivated species showed superior grain yield related agronomic performance as compared to the wild *Lens* species. But, several useful yield attributing traits like high number of pods plant^−1^, high number of seeds plant^−1^, high number of branches plant^−1^, and greater plant height as well as resistance to major foliar diseases was observed in various wild annual *Lens* species.

**Table 3:** Range of variations for different quantitative agro-morphological traits in annual *Lens* species.

Character	*Lens culinaris* ssp. *culinaris*			*Lens culinaris* ssp. *orientalis*			*Lens culinaris* ssp. *odemensis*		
	Range	Mean ± SE	CV (%)	Range	Mean ± SE	CV (%)	Range	Mean ± SE	CV (%)
**Days to 50% flowering**	34.50–43.00	39.75±1.89	9.53	27.00–135.00	93.76±2.35	32.81	88.00–143.00	126.42±1.81	9.30
**Days to maturity**	72.00–86.00	79.50±2.93	7.38	76.00–168.00	130.15±1.97	19.80	124.00–174.00	155.02±1.50	6.29
**Plant height (cm)**	17.00–24.00	20.50±1.55	15.12	12.33–95.50	26.58±0.68	33.48	20.33–59.94	33.38±1.59	30.94
**No. of branches plant^−1^**	6.50–9.50	8.12±0.80	19.70	4.00–35.00	13.47±0.68	66.07	3.50–22.00	14.69±0.32	29.06
**No. of pods plant^−1^**	18.50–208.00	92.50±44.50	96.22	3.00–250.00	59.58±3.68	80.89	5.50–185.50	74.75±6.95	60.29
**No. of seeds pod^−1^**	1.51–1.83	1.63±0.07	8.58	0.74–2.33	1.41±0.02	23.40	1.00–1.96	1.78±0.03	12.35
**No. of seeds plant^−1^**	28.50–316.50	147.37±67.62	91.76	4.00–409.00	82.53±5.1	80.84	6.00–357.50	136.26±13.83	65.77
**100-seed weight (g)**	0.75–2.50	1.62±0.38	47.53	0.15–4.00	0.91±0.03	56.04	0.40–1.10	0.73±0.02	24.65
**Seed yield plant^−1^ (g)**	0.25–4.25	1.85±0.89	96.75	0.01–3.26	0.80±0.04	80.00	0.06–3.30	1.05±0.11	68.57
**Biological yield plant^−1^ (g)**	6.35–13.90	8.92±1.70	38.22	0.50–18.50	3.73±0.15	54.95	0.25–10.55	5.45±0.29	35.59

**Table 4:** Range of variations for different quantitative agro-morphological traits in annual *Lens* species.

Character	*Lens culinaris* ssp. *tomentosus*			*Lens nigricans*			*Lens ervoides*			*Lens lamottei*		
	Range	Mean ± SE	CV (%)	Range	Mean ± SE	CV (%)	Range	Mean ± SE	CV (%)	Range	Mean ± SE	CV (%)
**Days to 50% flowering**	120.00–132.00	129.65±0.66	2.28	92.00–139.00	123.88±2.60	12.40	60.00–141.00	118.88±1.99	18.67	92.00–130.00	119.88±5.29	13.24
**Days to maturity**	20.00–173.00	162.75±0.78	2.16	130.00–176.00	160.42±2.41	8.90	93.00–173.00	153.63±1.97	14.38	130.00–165.00	154.11±4.65	9.05
**Plant height (cm)**	20.75–50.00	33.27±1.85	24.94	19.66–67.00	40.23±1.95	28.66	2.00–59.00	37.51±0.94	28.15	27.25–54.00	40.90±2.99	21.95
**No. of branches plant^−1^**	10.50–19.50	15.26±0.60	17.62	2.00–22.75	13.57±0.85	37.14	4.25–52.00	16.99±1.67	109.53	6.00–19.80	14.20±1.54	32.53
**No. of pods plant^−1^**	4.50–218.00	74.05±11.50	68.84	5.50–153.00	58.30±7.58	76.92	3.00–1002.00	111.78±11.74	116.94	8.00–185.50	63.97±18.80	88.19
**No. of seeds pod^−1^**	1.00–1.97	1.70±0.06	17.05	1.00–2.00	1.64±0.04	14.63	1.00–2.22	1.72±0.02	14.53	1.68–2.10	1.85±0.04	7.02
**No. of seeds plant^−1^**	6.00–420.00	133.00±22.30	74.97	7.50–275.00	100.45±14.01	82.46	3.00–1425.00	196.31±18.72	106.16	13.75–364.00	121.10±36.89	91.38
**100-seed weight (g)**	0.10–1.10	0.69±0.06	44.92	0.25–1.20	0.57±0.03	38.59	0.10–2.00	0.49±0.02	51.02	0.60–1.85	0.92±0.15	48.91
**Seed yield plant^−1^ (g)**	0.01–3.30	1.01±0.17	79.20	0.02–2.30	0.62±0.09	93.54	0.04–5.70	0.97±0.09	104.12	0.10–2.75	0.99±0.29	89.89
**Biological yield plant^−1^ (g)**	1.90–10.00	5.79±0.40	31.43	1.70–15.30	5.37±0.47	52.32	1.20–29.50	5.87±0.35	66.78	3.65–9.90	5.55±0.71	38.55

### Screening against foliar biotic stresses

#### Rust and powdery mildew

Many of the wild *Lens* accessions were resistant and moderately resistant to rust and powdery mildew during all the cropping seasons. Among accessions ILWL247 of *L*. *culinaris* ssp. *orientalis*; ILWL165, ILWL167 and ILWL238 of *L. culinaris* ssp. *odemensis*; ILWL37, ILWL22 and ILWL38 of *L. nigricans*; and ILWL40, ILWL41, ILWL54 and ILWL58 of *L. ervoides* recorded a score of 2 and identified as highly resistant to rust. While, ILWL350, ILWL369, and ILWL381 of *L. culinaris* ssp. *orientalis*; ILWL39 and ILWL81 of *L. culinaris* ssp. *odemensis*; ILWL191 of *L. nigricans,* and ILWL418, ILWL398, ILWL292, ILWL91 and ILWL294 of *L. ervoides* were found resistant to powdery mildew. Some of the accessions viz., ILWL37, ILWL38, ILWL22 and ILWL191 of *L. nigricans*, and ILWL269 of *L. ervoides* were rated as resistant to both rust and powdery mildew.

### Development of core set

“PowerCore” statistical programme selected a set of 96 accessions (24% of the entire collection), when data on both quantitative and qualitative variables were used in combination (Table 5). A total of 86 accessions (21%) were selected, when data only on quantitative variables were used, whereas 36 accessions (about 9%) could be selected, when data only on qualitative characters were used for analysis. Compared with the whole collection, the Shannon Diversity Indices (SDIs) were always higher in the core sets derived from different data sets indicating better representation of the existing diversity in each core set. Better representation of diversity was observed using “PowerCore” as compared to Principal Components Score Strategy (PCSS, Table 6).

**Table 5:** Species-wise representation of entire *Lens* collection and representation of accessions in core sets derived from different data sets.

*Lens* species	Accessions in entire collection	Accessions in core sets		
		I	II	III
*L. culinaris* ssp. *culinaris*	4	4	4	3
*L*. *culinaris* ssp. *orientalis*	171	21	19	7
*L*. *culinaris* ssp. *odemensis*	42	15	12	4
*L*. *culinaris* ssp. tomentosus	20	12	9	6
*L*. *nigricans*	35	18	17	7
*L*. *ervoides*	124	20	19	5
*L*. *lamottei*	9	6	6	4
Total	405	96 (23.7%)	86 (21%)	36 (8.9%)

Core sets based on I = combined data on both quantitative and qualitative variables; II = data only on quantitative variables, and III = data only on qualitative variables, respectively.

**Table 6:** Comparison of SDI of entire collection to core sets derived from different data sets.

S. No.	Traits	Entire collection	Core sets (Using PowerCore)*			Core set derived through PCSS
			I	II	III	
**Quantitative characters**						
1	Days to flowering	0.733	0.819	0.843	0.750	0.691
2	Days to maturity	0.795	0.851	0.881	0.685	0.729
3	Plant height	0.964	0.777	0.725	0.874	0.664
4	No. of branches plant^−1^	0.035	0.116	0.127	0.987	0.116
5	No. of pods plant^−1^	0.200	0.406	0.440	0.760	0.511
6	No. of seeds plant^−1^	0.297	0.533	0.635	0.632	0.716
7	No. of seeds pod^−1^	0.806	0.817	0.830	0.958	0.837
8	100-seed weight	0.725	0.883	0.914	0.766	0.710
9	Seed yield plant^−1^	0.735	0.745	0.698	0.816	0.698
10	Biological yield plant^−1^	0.637	0.972	0.641	0.787	0.821
11	Resistance to rust	0.942	0.979	0.973	0.849	0.908
12	Resistance to powdery mildew	0.886	0.953	0.948	0.880	0.843
**Qualitative characters**						
13	Seedling stem pigmentation	0.475	0.613	0.613	0.687	0.525
14	Leaf pubescence	0.963	0.917	0.952	0.966	0.982
15	Leaflet size	0.871	0.926	0.858	0.935	0.890
16	Tendril length	0.614	0.584	0.581	0.654	0.562
17	Flower colour	0.235	0.391	0.359	0.572	0.311
18	Ground colour of testa	0.543	0.679	0.629	0.787	0.562
19	Pattern of testa	0.790	0.915	0.880	0.970	0.805
20	Cotyledon colour	0.500	0.621	0.622	0.637	0.562
21	Pod shedding	0.956	0.833	0.819	0.921	0.809
22	Pod dehiscence	0.837	0.835	0.833	0.870	0.970

Core sets based on I = combined data on both quantitative and qualitative variables; II = data only on quantitative variables, and III = data only on qualitative variables, respectively.

### Diversity analysis of global *Lens* collection

**Figure 1:**
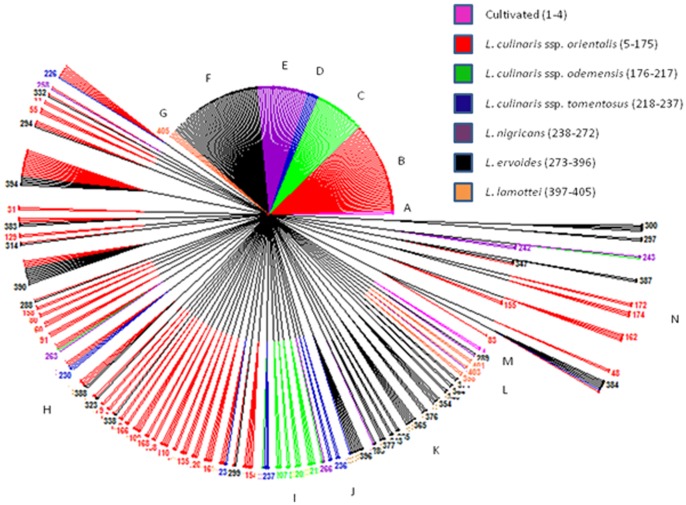
Hierarchical clustering of *Lens* accessions based on qualitative phenotypic data.

**Figure 2:**
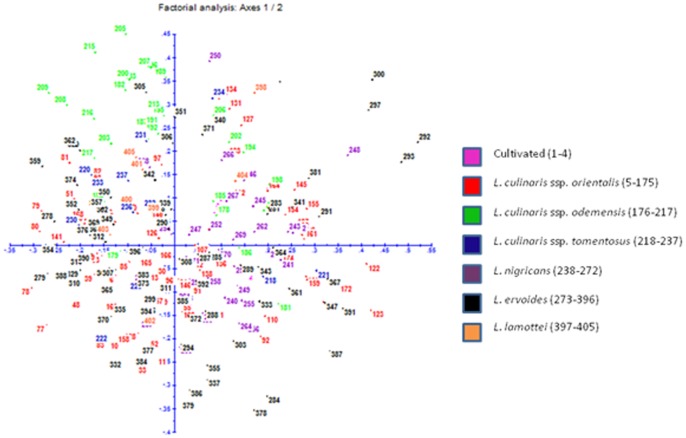
Factorial analysis of *Lens* accessions based on qualitative phenotypic data.

A Dice similarity matrix was generated based on the morphological qualitative data, which was further used to construct phylogenetic tree for deciphering the diversity of wild *Lens* species. Based on the Dice dissimilarity matrix, an average dissimilarity coefficient value of 0.5 was recorded. The dissimilarity coefficient ranged from 0.0–1.0 indicating that accessions showed 100% similarity to 100% dissimilarity (Table S1). Maximum dissimilarity was assessed between ILWL429 (*L. lamottei*, Spain) and ILL10829 (*L. culinaris* ssp. *culinaris*, Syria); ILWL126 (*L. ervoides*, Syria) and ILL10829 (*L. culinaris* ssp. *culinaris,* Syria), whereas ILWL65 (*L. ervoides*, Turkey) showed 100% dissimilarity to the accessions of *L. culinaris* ssp. *orientalis*, namely ILWL378 (Turkmenistan), ILWL241 (Syria), ILWL231 (Syria) and ILWL230 (Syria); ILWL55 (*L. ervoides*, Israel) to *L. culinaris* ssp. *orientalis* accessions namely ILWL378 (Turkmenistan), ILWL241 (Syria), ILWL231 (Syria) and ILWL230 (Syria); ILWL55 (*L. ervoides*, Israel) to ILWL378 (*L. culinaris* ssp. *orientalis*, Turkmenistan); EC718264 (*L. nigricans*, Spain) to ILWL238 (*L. culinaris* ssp. *odemensis*, Syria); ILWL23 (*L. nigricans*, Italy) to ILWL241 (*L. culinaris* ssp. *orientalis*, Syria); ILWL97 (*L. culinaris* ssp. *tomentosus,* Turkey ) to ILWL125 (*L. culinaris* ssp. *orientalis,* Syria); ILL10829 (*L. culinaris* ssp. *culinaris,* Syria) to *L. culinaris* ssp. *odemensis* from Syria including ILWL357, ILWL238 and ILWL167; ILWL371 (*L. culinaris* ssp. *orientalis*, Syria) to *L. culinaris* ssp. *orientalis* ILWL62 (11) and ILWL8 (9); ILWL227 (*L. culinaris* ssp. *orientalis*, Syria) to accessions of the same species ILWL62 (11) and ILWL8 (9). After exclusion of accessions, which showed 100% similarity to each other with dissimilarity matrix value of 0.0 (Table S1), the minimum dissimilarity was exhibited by ILWL247 (*L. culinaris* ssp. *orientalis,* Syria) to *L. ervoides* namely EC718432 (HRV), EC718429 (HRV), EC718427 (Ukraine), EC718422 (Turkey), ILWL67 (Turkey), and ILWL121 (*L. culinaris* ssp. *tomentosus*, Syria); ILWL443 (*L. culinaris* ssp. *orientalis,* Turkey) to other accessions of *L. culinaris* ssp. *orientalis,* ILWL469 (Syria), ILWL416 (Syria), ILWL402 (Lebanon), ILWL385 (Uzbekistan) and ILWL384 (Tajikistan); ILWL247 (*L. nigricans*, Syria) to *L. culinaris* ssp. *orientalis* accessions ILWL86 (Turkey), ILWL95 (Turkey) and ILWL117 (Syria), with dissimilarity coefficient of 0.053. The hierarchical clustering of accessions resulted into various clusters A–N, where clusters A–G, J and M were occupied by accessions of respective individual species, whereas other clusters included accessions from two or more species as shown in Fig. 1. The grouping pattern of different accessions in hierarchical clustering is listed along with their species and respective groups (Table S2). Clustering pattern of the various accessions obtained through factorial analysis, where first and second principal coordinates explained only 10.17% and 8.8% variations, respectively (Fig. 2). In general, the clustering pattern of accessions, in both the hierarchical and factorial analysis was not according to their geographical origin.

**Figure 3:**
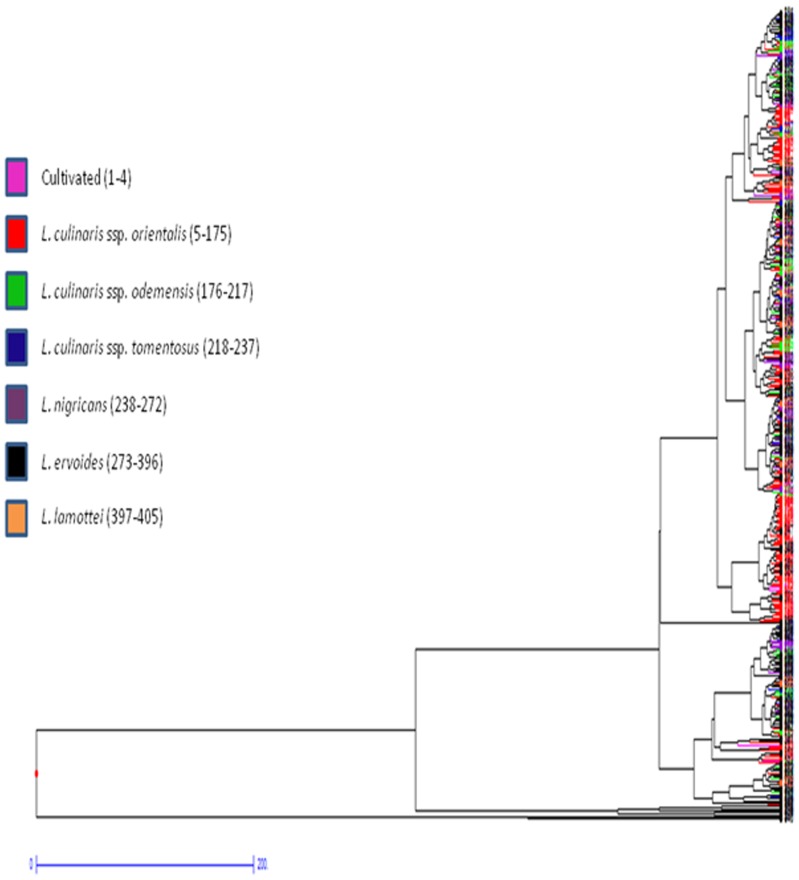
Hierarchical clustering of *Lens* accessions based on quantitative phenotypic data.

**Figure 4:**
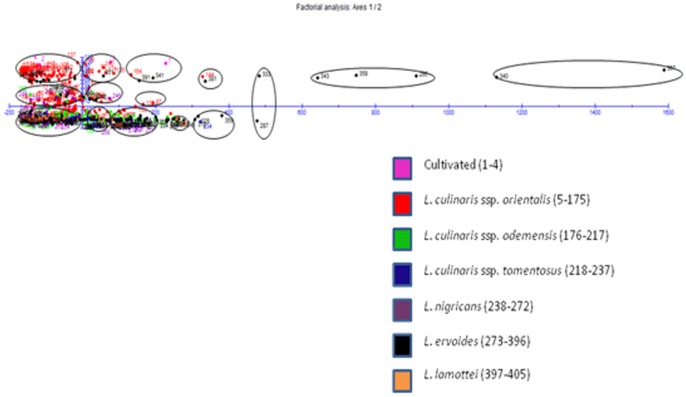
Factorial analysis of *Lens* accessions based on quantitative phenotypic data.

In case of quantitative data, the Euclidean dissimilarity matrices varied from 2.67 between ILWL159 (Syria) and ILWL67 (Turkey) to 1740.53 between ILWL418 (Syria) and ILWL276 (Turkey) of *L. ervoides* (Table S3). In hierarchical clustering, two major clusters were formed in which major cluster I was occupied by only two accessions namely ILWL292 and ILWL418 of *L. ervoides* from Turkey and Syria, respectively, whereas cluster II included others. Major cluster II further divided into two clusters A and B, where A was occupied by accessions of *L. ervoides*, ILWL401 (Lebanon), ILWL321, ILWL58 and ILWL269 (Turkey), and ILWL51 (Montenegro), whereas cluster B was occupied by accessions from all seven species included in the study (Fig. 3). Cluster B was further divided into sub-clusters (B1 and B2), groups (a, b, c and d) outgroups (a1 and a2, b1 and b2, d1 and d2), clutches (b2a and b2b, d1a and d1b, d2a and d2b) and finally to sub-clutches (i-xxiii) as shown in Fig. 3 (Table S4). Cluster II was reported to be more heterogeneous with different sub-clusters and few individual accessions forming separate groups. Factorial analysis verified the results obtained *via* hierarchical clustering as it resulted into almost similar clustering pattern as shown in Fig. 4. In factorial analysis, first two principal coordinates explained 98.26 per cent cumulative variations with 92.94 and 5.32 per cent dissimilarity, respectively. The clustering pattern in both, the hierarchical and factorial analysis, grouping of accessions was not according to their geographical origin.

### Diversity analysis of *Lens* core collection

**Figure 5:**
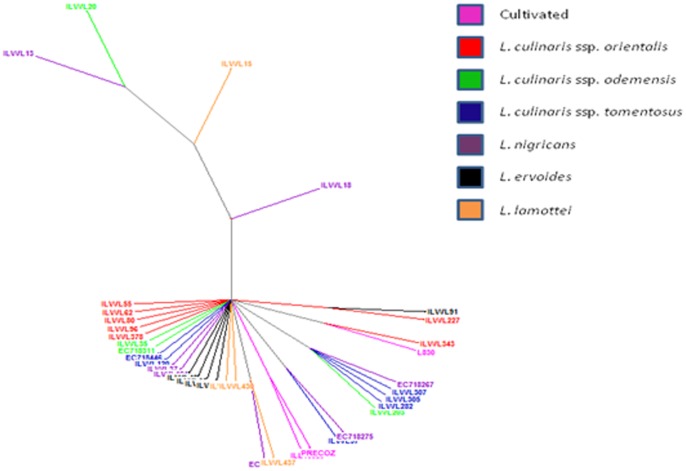
Hierarchical clustering of *Lens* core set accessions based on qualitative phenotypic data.

**Figure 6:**
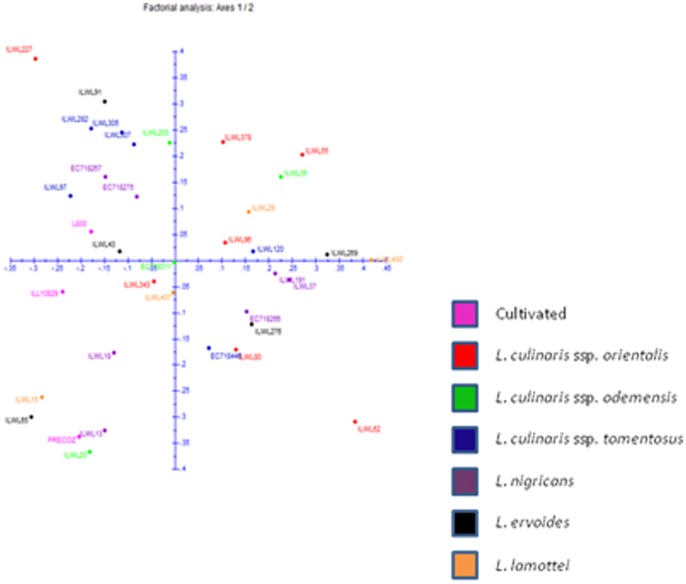
Factorial analysis of *Lens* core set accessions based on qualitative phenotypic data.

Based on Dice dissimilarity matrix, the maximum dissimilarity matrix values ranged from 0.1–1.0 with an average of 0.55. No two genotypes showed 100% similarity to each other (Table S5). Minimum diversity was observed between accessions ILWL13 (*L. nigricans*, Italy) and ILWL20 (*L. culinaris* ssp. *odemensis*, Palestine); ILWL437 (*L. lamottei*, Turkey) and EC718266 (*L. nigricans*, Italy), with dissimilarity matrix value of 0.1. Maximum dissimilarity i.e. 100% was found between ILWL65 (*L. ervoides*, Turkey) and ILWL378 (*L. culinaris* ssp. *orientalis,* Turkmenistan); and ILWL227 (*L. culinaris* ssp. *orientalis*, Syria) and ILWL62 (*L. culinaris* ssp. *orientalis*, Turkey). In hierarchical clustering of the accessions, ILWL20 (*L. culinaris* ssp. *odemensis*, Palestine), ILWL13 (*L. nigricans*, Italy), ILWL15 (*L. lamottei,* Turkey) and ILWL18 (*L. nigricans*, France) each constitute a separate group alone as shown in Fig. 5. EC718266 of *L. nigricans* (Italy) clustered with ILWL437 of *L. lamottei* (Turkey), whereas ILWL97 of *L. culinaris* ssp. *tomentosus* (Turkey) clustered with EC718275 of *L. nigricans* (Turkey). ILWL203 (*L. culinaris* ssp. *odemensis*, Turkey), ILWL282, ILWL305 and ILWL307 (*L. culinaris* ssp. *tomentosus*, Turkey), and EC718267 (*L. nigricans*, Montenegro) occupied the same cluster. Further, ILWL343 and ILWL227 from *L. culinaris* ssp. *orientalis* (Syria) formed group with L830 of *L. culinaris* ssp. *culinaris* (India) and ILWL91 of *L. ervoides* (Turkey), respectively. Factorial analysis was also carried out as shown in Fig. 6, where PC1 and PC2 explained only 12.99 and 12.53% variations respectively. In general, first 12 principal coordinates had Eigen values >1.0. The clustering pattern in both, hierarchical and factorial analysis, did not reflect their geographical origin.

**Figure 7:**
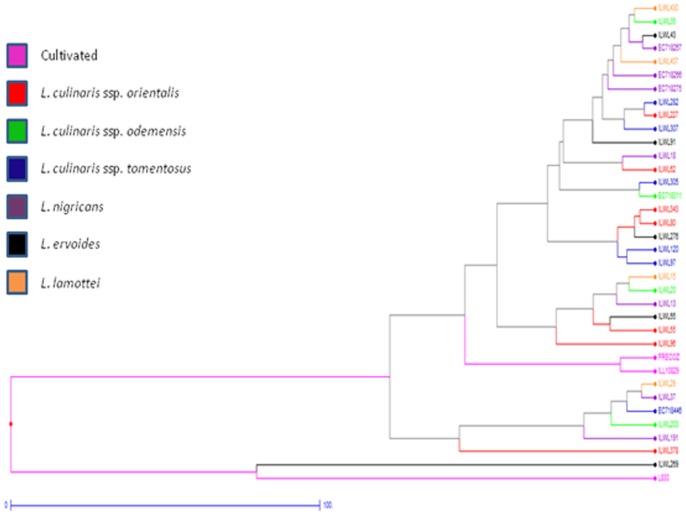
Hierarchical clustering of *Lens* core set accessions based on quantitative phenotypic data.

**Figure 8:**
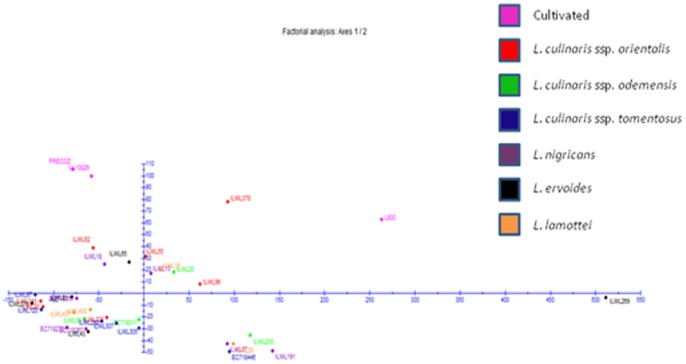
Factorial analysis of *Lens* core set accessions based on quantitative phenotypic data.

In case of quantitative data, the Euclidean dissimilarity matrices ranged from 7.15 between accessions ILWL282 (*L. culinaris* ssp. *tomentosus*, Turkey) and ILWL227 (*L. culinaris* ssp. *orientalis*, Syria) to 635.85 between accessions ILWL276 and ILWL269 of *L. ervoides* from Turkey (Table S6). The hierarchical clustering of accessions, where all the outgroups were occupied by accessions from different species excepting one containing Precoz (*L. culinaris* ssp. *culinaris*, Brazil) and ILL10829 (*L. culinaris* ssp. *culinaris,* Syria) as shown in Fig. 7. Factorial analysis resulted into first two principal coordinates having Eigen values >1 where first and second principal coordinates explained 89.6 and 8.85% variability respectively (Fig. 8). The clustering pattern in both, hierarchical and factorial analysis was not according to their geographical origin.

## Discussion

Crop wild relatives (CWRs) can contain far greater diversity than their domestic counterparts and may hold increased potential to adapt against crop pests and diseases, adverse weather conditions and longer term changes in climate. One way to improve the resilience of our crop plants is to harness the genetic diversity found in their wild relatives. By introducing CWRs into breeding programs, the useful traits they contain, such as high yield and disease resistance, can be passed into cultivated crops. The introduction, characterization, evaluation, maintenance and utilization of CWRs for important morphological and agronomic traits, including adaptation to biotic and abiotic stresses, are often prerequisites for conducting a successful crop breeding research in annual plant species with a view to introgress novel trait of interest [39]–[41]. Effective evaluation and screening of diverse germplasm, especially unadapted gene pool [42]–[43] may prove useful to retrieve invaluable phenotypes to combat emerging agricultural challenges [44]. A strong pre-breeding program is needed to introgress genes from distant species and supply breeding pools with adequate parental germplasm.

### Species characterization and agronomic evaluation

The results of the present study showed that global wild annual *Lens* collection of diverse origin had significant variation with respect to morphological and agronomic characters. Most of the wild annual *Lens* species had wide variations to seedling stem pigmentation and low to dense leaf pubescence. Dense leaf pubescence character can contribute towards better tolerance to insects and is a subject of further detailed investigation. More importantly to note that variation was observed within a species for leaflet size, tendril length, pod shedding, pod dehiscence, flower colour, ground colour of testa, pattern of testa and cotyledon colour (Table 2). These morphological plant characteristics can help to distinguish distinct phenotypic classes and their mode of inheritance can be studied using principles of classical genetics. Some of these traits are considered as descriptors for the taxonomic classification of taxon into their respective species and sub-species. It also allows phenotypic identification of specific alleles for specific loci [45]–[46]. Further, there were substantial variations exhibited in *Lens* species for important agro-morphological traits as reflected by their range, mean and coefficient of variation (Table 3 and 4), suggesting that it could be due to the diverse origin of these species and to their taxonomic identification. However, seed yield plant^−1^ and biological yield plant^−1^ were higher in magnitude in the cultivated varieties, including Precoz, L830, ILL10829 and ILL8006 in both the agro-climatic regions. By contrast, in wild annual *Lens* species, the range and mean performance found promising for useful agro-morphological traits, including high number of pods plant^−1^ in accessions of *L. ervoides* viz., ILWL418 (1002 pods plant^−1^), collected from Syria and ILWL321 (403 pods plant^−1^), ILWL401(478 pods plant^−1^), ILWL292 (697 pods plant^−1^), ILWL369 (355 pods plant^−1^) and ILWL308 (218 pods plant^−1^) collected from Turkey. Other important accessions for high number of pods plant^−1^ and resistance to rust and powdery mildew in *L. nigricans* included, ILWL191 (141 pods plant^−1^), ILWL40 (113 pods plant^−1^), ILWL41 (147 pods plant^−1^) and ILWL42 (143 pods plant^−1^), also collected from Turkey. It is important to note that, wild accessions collected from Turkey exhibited substantial variations for useful agro-morphological traits including multiple resistance against major foliar diseases. These promising accessions of *L. nigricans* and *L. ervoides* from the secondary genepool can be exploited for broadening the genetic base of cultivated lentil. Further, rust and powdery mildew resistance gene sources identified can be of great significance for exploiting in breeding resistant lentil varieties, especially for the epidemiologically important regions in the lentil growing areas of the world. The list of some important accessions selected from different *Lens* species carrying useful traits of interest is presented in Table 7. The present study further strongly suggests revisiting of Turkey and other areas for more collections through explorations. In order to make quality *ex-situ* collection, it is necessary to know the geographical distribution of the target *Lens* taxa and where the gaps in the seed collection occur. Knowledge of current ICARDA genebank holding and understanding the gaps in collection would help prioritize the species and locations of wild *Lens*, that are in most need of collecting and conserving them in gene banks.

**Table 7:** Identification of promising accessions for few agronomic and major biotic traits for their introgression in lentil enhancement selected from the entire *Lens* collection.

S. No.	Accessions	Species	Gene pool	Trait of Interest	Origin
1.	ILWL 247, 255, 476,	*L. culinaris* ssp. *orientalis*	Primary	High number of pods plant^−1^, resistant to rust	Syria, Turkey
2.	ILWL 346, 350, 369, 378, 490	*L. culinaris* ssp. *orientalis*	Primary	High number of pods plant^−1^, resistant to powdery mildew	Syria, Armenia
3.	ILWL 372	*L. culinaris* ssp. *orientalis*	Primary	High number of pods plant^−1^, resistant to rust and powdery mildew	Uzbekistan
4.	ILWL 80, 381	*L. culinaris* ssp. *orientalis*	Primary	Resistant to rust and powdery mildew	Tajikistan
5.	ILWL 456	*L. culinaris* ssp. *orientalis*	Primary	High 100-seed weight and resistant to rust	Turkey
6.	ILWL 39	*L. culinaris* ssp. *odemensis*	Primary	Resistant to rust and powdery mildew	Turkey
7.	ILWL 165, 203, 237, 238	*L. culinaris* ssp. *odemensis*	Primary	High number of pods plant^−1^, resistant to rust	Syria
8.	ILWL 167	*L. culinaris* ssp. *odemensis*	Primary	Resistant to rust and powdery mildew	Syria
9.	ILWL 97	*L. culinaris* ssp. *tomentosus*	Primary	Resistant to powdery mildew	Turkey
10.	ILWL 121, EC-718446	*L. culinaris* ssp. *tomentosus*	Primary	High number of pods plant^−1^	Syria
11.	ILWL 23, 24, 25	*L. nigricans*	Secondary/Tertiary	Resistant to rust	Italy, HRV
12.	ILWL 22	*L. nigricans*	Secondary/Tertiary	Resistant to rust and powdery mildew	Italy
13.	ILWL 37, 191	*L. nigricans*	Secondary/Tertiary	High number of pods plant^−1^, resistant to rust and powdery mildew	Turkey
14.	ILWL 40, 41, 42, 159, 269, 292,294, 321, 398, 418, EC-718428, 718439	*L. ervoides*	Secondary/Tertiary	High number of pods plant^−1^, resistant to rust and powdery mildew	Ukraine, Turkey, Italy, Syria,
15.	ILWL 50, 54, 56, 58	*L. ervoides*	Secondary/Tertiary	High number of pods plant^−1^, resistant to rust	HRV, Israel, PSE, Turkey
16.	ILWL 91	*L. ervoides*	Secondary/Tertiary	Resistant to powdery mildew	Turkey
17.	ILWL 29, EC-718692	*L. lamottei*	Secondary/Tertiary	High number of pods plant^−1^	Spain, France

Assessment of among and within population diversity will also help to establish the potential of *in-situ* conservation of wild *Lens* species. Many areas of greatest interest for *in- situ* conservation (e.g. Turkey and other Mediterranean countries) are suffering from rapid loss of invaluable genetic resources due to habitat destruction [47]. Important areas to target for *in-situ* conservation includes west Turkey for *L. nigricans*, Southwest Turkey, North-west Syria, south Syria and Jordan for *L. culinaris* spp. *orientalis*, south Syria for *L. culinaris* ssp. *odemensis* and the coastal border region between Turkey and Syria stretching along the Syrian coast for *L. ervoides* [48]. The accumulated information on this global collection of wild *Lens* will be used to conduct a gap analysis using appropriate approaches to guide future collecting missions to fill the gaps, but also to conduct traits-targeted collecting missions to sample new accessions with potential resistance/tolerance to major biotic and abiotic stresses. The same information can be used to define natural habitats that can allow effective *in-situ* conservation of the species richness as well as populations with potential sought traits. Maxted et al. (2012b) [49] proposed the Fertile Crescent, mainly the Aegean and South-western region of Turkey, North Syria and Lebanon as appropriate sites for *in-situ* conservation of most of temperate legume species.

### *Lens* core collection

“PowerCore” is a rapid approach for developing core collection, which effectively simplifies the generation process of core set with reduced number of core entries, but maintaining high percent of diversity as compared to other methods used [22], [50]. For larger genebank holdings, the core sets identified using “PowerCore” normally are small in size with greater diversity captured as compared to traditional clustering procedures. However, in the present study, as the number of accessions representing different *Lens* taxa in the entire collection were low, relatively more number of accessions were selected when quantitative variables were used either in combination with qualitative characters or alone. Using qualitative variables alone, a smaller core size of about 9% was achieved.

Majority of the wild *Lens* taxa in the entire collection belongs to Syria (37%) and Turkey (28%). However, maximum representation of accessions in core collections, derived from all data sets was from Turkey, followed by Syria, indicating rich diversity for many of the wild relatives of cultivated lentil. It is only in Aegean and the South-western region that the distribution of all four wild taxa of the genus *Lens* overlaps [51]. Unfortunately, Turkey, like other Mediterranean countries is suffering the rapid loss of many of its valuable genetic resources. The Aegean and South-western region of Turkey is, therefore, particularly important for exploration and collection of wild taxon for conservation *ex-situ* and also for analysis of within population variations in order to assess the threat of genetic erosion to each species and potential for *in-situ* conservation. Ferguson et al. (1996) [51] reported that *L. culinaris* ssp. *odemensis* and *L. ervoides* are the taxa most threatened by genetic erosion. Selection of core entries using different data sets gives a choice to the users as representation of common accessions across different core sets varies with the data sets used. A total of 66 and 25 accessions were common between the core sets developed using combined data on qualitative and quantitative variables and that obtained from quantitative and qualitative variables alone, respectively. Only 15 accessions were common between the core set obtained from quantitative and qualitative variables separately. The user, therefore, has a choice to use his experience and decide which core sets better serves the specific purpose.

Of the 46 accessions identified as promising for various desired traits from the entire *Lens* accessions (Table 7), a total of 21, 15 and 10 accessions, respectively, were represented in core sets derived from different data sets i.e. quantitative and qualitative traits combined, and quantitative and qualitative traits individually. Representation of proportionately greater number of desired accessions in the core set derived from qualitative morphological data could be the existence of good polymorphism with different descriptor states for qualitative traits with major gene effect. The core sets derived from different data sets gives us an opportunity to precisely estimate diversity at genetic/molecular level for use by the germplasm curators and the users. As majority of accessions in the core set derived from qualitative data originate from Turkey, this indirectly indicates greater polymorphism occurring in accessions originating from parts of Turkey (Table 8). Therefore, it is imperative that the wild gene pool of Turkey needs to be further explored, collected, conserved, characterized, and used in crop improvement. As has been emphasized, for broadening the genetic base of crop productions, where domestication is the bottleneck, protecting the wild gene pool is an utmost necessity in order to reconstructing the early evolutionary stages [52] to facilitate evolution. When selecting the species to conserve *in-situ*, the highest emphasis should be given to most genetically distinct groups of taxa (the widespread species with a wide range of adaptation). *L. culinaris* ssp. *orientalis* are distributed in Turkey throughout the northern belt of Fertile Crescent and share different types of habitat in different ecosystems, and they have a wide range of adaptability [53]. A few promising common accessions found across all core sets include ILWL29 (*L. lamottei*) from Spain; ILWL37 (*L. nigricans*) from Turkey; ILWL97 (*L. culinaris* ssp. *tomentosus*) from Turkey; ILWL269 (*L. ervoides*) from Turkey, and ILWL378 (*L. culinaris* ssp. *orientalis*) from Syria (Table 9). Besides possessing disease resistance, these accessions were also promising for yield related traits as high number of pods plant^−1^ and have significant breeding potential for yield enhancement.

**Table 8:** Agro-ecological representation of species-wise accessions in entire *Lens* collection and core sets derived from different data sets.

S. No.	Origin	Accessions in entire collection	Accessions in core sets		
			I	II	III
1	Alpes-Cote d’Azur	1	1	1	–
2	Armenia	2	1	1	–
3	Azerbaijan	1	–	–	–
4	Bahrain	4	–	1	–
6	Cyprus	3	–	–	–
7	Former Soviet Union	2	–	–	–
8	Former Yugoslavia	2	–	–	–
9	France	3	3	2	1
10	HRV	14	2	2	1
11	Iran	2	–	–	–
12	Israel	10	2	1	1
13	Italy	8	2	3	2
14	Jordan	11	1	1	–
15	Lebanon	9	2	2	–
16	Montenegro	3	–	1	1
17	Palestine	1	1	–	1
18	PSE	3	–	–	–
19	Russia	1	–	–	–
20	Spain	12	6	5	2
21	Syria	152	20	24	3
22	Tajikistan	4	2	1	–
23	Turkmenistan	2	1	1	1
24	Turkey	113	28	22	14
25	UBA	1	–	–	–
26	Ukraine	3	1	1	–
27	Uzbekistan	8	2	–	–
28	Unknown	30	21	17	9
	**Total**	**405**	**96**	**86**	**36**

**Table 9:** Identification of some promising accessions for few agronomic and major biotic traits for their introgression in lentil enhancement selected from the qualitative core set.

S. No.	Accessions	Species	Gene pool	Trait of Interest	Origin
1.	ILWL 80	*L. culinaris* ssp. *orientalis*	Primary	Resistant to rust and powdery mildew	Tajikistan
2.	ILWL 378	*L. culinaris* ssp. *orientalis*	Primary	High number of pods plant^−1^, resistant to powdery mildew	Turkmenistan
3.	ILWL 203	*L. culinaris* ssp. *odemensis*	Primary	High number of pods plant^−1^, resistant to rust	Syria
4.	EC-718446	*L. culinaris* ssp. *tomentosus*	Primary	High number of pods plant^−1^	Syria
5.	ILWL 97	*L. culinaris* ssp. *tomentosus*	Primary	Resistant to powdery mildew	Turkey
6.	ILWL 37	*L. nigricans*	Secondary/Tertiary	High number of pods plant^−1^, resistant to rust and powdery mildew	Turkey
7.	ILWL 191	*L. nigricans*	Secondary/Tertiary	High number of pods plant^−1^, resistant to rust and powdery mildew	Turkey
8.	ILWL 91	*L. ervoides*	Secondary/Tertiary	Resistant to powdery mildew	Turkey
9.	ILWL 269	*L. ervoides*	Secondary/Tertiary	High number of pods plant^−1^, resistant to rust and powdery mildew	Turkey
10.	ILWL 29	*L. lamottei*	Secondary/Tertiary	High number of pods plant^−1^	Spain

### Diversity assessment

The present study depicted a wide genetic variation in the wild annual *Lens* collection for agro-morphological traits as revealed by the dendrogram generated from the dissimilarity matrix and factorial analysis using DARwin 5 approach. In case of entire collection, qualitative data analysis revealed that maximum variability among accessions was interspecific and not limited to a particular country and this could be partly explained by the taxonomic differences. But in case of quantitative data, minimum as well as maximum diversity was reported among *L. ervoides* accessions collected from Turkey and Syria. Among core set accessions, qualitative data analysis revealed maximum interspecific as well as intraspecific variability between *L. ervoides* and *L. culinaris* ssp. *orientalis,* and among *L. culinaris* ssp. *orientalis*, accessions, respectively. Further, quantitative data revealed maximum diversity among *L. ervoides* accessions from Turkey. The diversity could mainly be attributed to diverse agro-ecological conditions as the current study included global wild annual *Lens* collection. The Euclidian distances calculated based on the quantitative data manifested large genetic distances among the accessions. Vieira et al. (2007) [54] analysed 19 wheat accessions for 17 phenotypic characters and reported genetic distances upto 196.61. A large genetic distance between heterotic germplasm can be useful for developing lines with good combining ability in heterosis breeding [55]–[56]. The knowledge of genetic diversity among accessions provides clues about the heterotic potential that can be exploited while making crosses in order to create polymorphic populations. The clustering of accessions on the basis of phenotypic multivariate data, as compared to the molecular classification, was observed to be a much better procedure to explain the genotypic effects, since, the percentage of sum of squares between phenotypic sub-populations was higher in magnitude than that of between genetic sub-populations for all the traits analysed [57].

When genetic diversity was compared based on qualitative traits between entire set and core set, the core set included accessions which explained reasonable variability. In case of wild taxa namely *L. culinaris* ssp. *culinaris*, *L. culinaris* ssp. *orientalis*, *L. culinaris* ssp. *odemensis* and *L. lamottei*, core set included representative accessions from each group categorised based on entire set qualitative data excluding two to three accessions from *L. nigricans* and *L. culinaris* ssp. *tomentosus,* each. Further, accessions of various species namely *L. culinaris* ssp. *odemensis* from Palestine, *L. nigricans* from Italy and France, and *L. lamottei* from Turkey geographical origins constituted separate groups alone. Thus overall, it can be suggested that based on qualitative data, core set accessions revealing substantial diversity as available in the entire global collection. In case of quantitative data, core set included accessions from all the species and from majority of outgroups formed in genetic diversity analysis of entire set. Wang et al. (2006) [58] developed a core set capturing the genetic diversity of large soybean collection using 2% of total accessions to represent about 70% of the diversity from a whole sample set.

The tree formed from Dice Co-efficient and Euclidean distances of the standardized phenotypic means showed that genetic relatedness rarely matched analogous geographical origins in general. Plants that are phenotypically similar are not necessarily genetically so, as different gene pools could result in similar phenotypes. The reason might be continuous gene flow among wild species occurring in nature. Among wild taxa, one important aspect for species prioritization is the degree of relatedness, as it determines the actual potential for introducing useful traits from the wild into cultigen. Relatedness information derived from a combination of biosystematics based upon traditional morphological and genotypic data, which are being used increasingly as well as information derived from plant breeders attempting crosses between CWRs and crops [59] are extremely useful for planning future study for the introgression of crop wild relatives.

## Conclusions

The present study has helped in identifying the useful gene sources viz, earliness, high number of branches plant^−1^, high number of pods plant^−1^ and, multiple disease resistance etc. in different *Lens* taxa across gene pools. Diversity analysis suggested ample scope for future germplasm collecting from the hotspots in Turkey and Syria. The promising trait-specific accessions reported particularly in *L. nigricans* and *L. ervoides* need to be considered while planning future lentil breeding programme for introgressing gene of interests from wild *Lens*. Further, certain accessions belonging to the secondary gene pool of *Lens* and also extracted in core set developed, can be used as a starting material aimed at large scale base broadening of cultivated lentil.

## Supporting Information

Table S1 **Dissimilarity matrices of** ***Lens*** **accessions based on qualitative morphological data.**
(XLSX)

Table S2 **Clustering of entire** ***Lens*** **collection based on qualitative morphological data.**
(DOCX)

Table S3 **Dissimilarity matrices of** ***Lens*** **accessions based on quantitative agro-morphological data.**
(XLSX)

Table S4 **Clustering of entire** ***Lens*** **collection based on quantitative agro-morphological data.**
(DOCX)

Table S5 **Dissimilarity matrices of** ***Lens*** **accessions constituting the core set based on qualitative morphological data.**
(XLSX)

Table S6 **Dissimilarity matrices of** ***Lens*** **accessions constituting the core set based on quantitative agro-morphological data.**
(XLSX)
