# Supplementary material for: Global Wild Annual Lens Collection: A Potential Resource for Lentil Genetic Base Broadening and Yield Enhancement
Source: PLoS One. 2014 Sep 25;9(9):e107781. doi: 10.1371/journal.pone.0107781 (PMC4177869; doi:10.1371/journal.pone.0107781)
Supplement: Table S2 — Clustering of entire Lens collection based on qualitative morphological data. (DOCX) [file pone.0107781.s002.docx]

**Table S2: Clustering of entire *Lens* collection based on qualitative morphological data.**

| **Group** | **Accessions** | **Species present** |
| --- | --- | --- |
| A | L830, ILL10829 | *L. culinaris* ssp. *culinaris* |
| B | ILWL4, ILWL55, ILWL69, ILWL70, ILWL78, ILWL80, ILWL87, ILWL88, ILWL93, ILWL101, ILWL122, ILWL125, ILWL143, ILWL177, ILWL227, ILWL228, ILWL241, ILWL244, ILWL246, ILWL249, ILWL286, ILWL290, ILWL297, ILWL301, ILWL324, ILWL341, ILWL346, ILWL353, ILWL354, ILWL355, ILWL358, ILWL359, ILWL360, ILWL367, ILWL371, ILWL373, ILWL374, ILWL378, ILWL380, ILWL386, ILWL447, ILWL456, ILWL466, ILWL475, ILWL476, ILWL477, EC718617 | *L. culinaris* ssp. *orientalis* |
| C | ILWL21, ILWL23, ILWL39, ILWL81, ILWL83, ILWL100, ILWL108, ILWL116, ILWL160, ILWL166, ILWL203, ILWL221, ILWL235, ILWL237, ILWL238, ILWL314, ILWL409, ILWL436, ILWL438, ILWL462, ILWL468, ILWL470, EC718311, EC718312, EC718693, EC718694 | *L. culinaris* ssp. *odemensis* |
| D | EC718446, ILWL97, ILWL120, ILWL282, ILWL305, ILWL308 | *L. culinaris* ssp. *tomentosus* |
| E | ILWL06, ILWL08, ILWL09, ILWL17, ILWL19, ILWL22, ILWL23, ILWL24, ILWL25, ILWL28, ILWL30, ILWL31, ILWL33, ILWL34, ILWL37, ILWL111, ILWL315, ILWL460, EC718264, EC718265, EC718267, EC718270, EC718271, EC718272, EC718273, EC718275 | *L. nigricans* |
| F | ILWL40, ILWL41, ILWL43, ILWL46, ILWL47, ILWL50, ILWL51, ILWL54, ILWL91, ILWL92, ILWL123, ILWL126, ILWL127, ILWL128, ILWL130, ILWL133, ILWL134, ILWL135, ILWL158, ILWL260, ILWL269, ILWL274, ILWL276, ILWL284, ILWL292, ILWL294, ILWL299, ILWL340, ILWL388, ILWL395, ILWL398, ILWL400, ILWL406, ILWL408, ILWL410, ILWL415, ILWL418, ILWL439, ILWL441, ILWL450, ILWL457, ILWL461, EC718425, EC718426, EC718428, EC718433, EC718434, EC718439, EC718440 | *L. ervoides* |
| G | ILWL29, ILWL430, ILWL437, EC718692 | *L. lamottei* |
| H | ILWL3, ILWL98, ILWL103, ILWL145, ILWL183, ILWL256, ILWL422, ILWL195, ILWL6, ILWL38, ILWL7, ILWL265, ILWL8, ILWL62, ILWL71, ILWL72, ILWL147, ILWL73, ILWL74, ILWL75, ILWL57, ILWL76, ILWL77, ILWL82, ILWL84, ILWL85, ILWL92, ILWL102, ILWL105, ILWL106, ILWL113, ILWL146, ILWL171, ILWL180, ILWL377, ILWL185, ILWL270, ILWL399, EC718441, EC718442 , ILWL79, ILWL89, ILWL81, ILWL104, ILWL148, ILWL323, EC718430, ILWL94, ILWL365, ILWL96, ILWL138, ILWL97, ILWL150, ILWL350, ILWL381, ILWL139, ILWL142, ILWL159, ILWL162, ILWL232, ILWL407, ILWL411, EC718437, EC718438, ILWL109, ILWL52, ILWL115, ILWL455, ILWL124,ILWL243, ILWL152, ILWL157A, ILWL157B, ILWL176, ILWL278, ILWL281, ILWL178, ILWL277, ILWL331, ILWL35, ILWL474, ILWL181, ILWL192, ILWL219, ILWL149, ILWL194, ILWL199, ILWL182, ILWL325, ILWL366, ILWL370, ILWL204, EC718436, ILWL201, ILWL220, ILWL184, ILWL225, ILWL242, ILWL230, ILWL231, ILWL248, ILWL280, ILWL253, ILWL364, ILWL255, ILWL478, ILWL293, ILWL295, ILWL309, ILWL310, ILWL302, ILWL484, ILWL312, ILWL376, ILWL313, ILWL317, ILWL330, ILWL332, ILWL326, ILWL329, ILWL335, ILWL342, ILWL372, ILWL344, ILWL345, ILWL349, ILWL347, ILWL348, ILWL444, ILWL467, ILWL369, ILWL485, ILWL489, ILWL307, ILWL382, ILWL64, ILWL417, ILWL423, ILWL424, ILWL425, ILWL426 | *L. culinaris* ssp. *orientalis*,  *L. culinaris* ssp. *tomentosus*,  *L. nigricans*,  *L. ervoides*,  *L. culinaris* ssp. *odemensis* |
| I | ILWL10, ILWL196, ILWL198, EC718673, ILWL165, ILWL254, ILWL464, ILWL167, ILWL357, ILWL222, ILWL361, ILWL362, ILWL320, EC718308, EC718309, EC718310, ILWL472, EC718266 | *L. culinaris* ssp. *odemensis*,  *L. culinaris* ssp. *tomentosus*,  *L. nigricans* |
| J | ILWL90, ILWL93, ILWL197, EC718672 | *L. culinaris* ssp. *tomentosus* |
| K | ILWL480, ILWL27, ILWL28, ILWL29, ILWL30, ILWL36, EC718448, EC718449, ILWL27, ILWL49, ILWL42, ILWL44, EC718424, ILWL58, ILWL321, ILWL63, ILWL271, ILWL131, ILWL141, ILWL155, ILWL156, ILWL234, ILWL414, ILWL137, ILWL334, EC718423, ILWL251, ILWL261, ILEL263, ILWL396, ILWL397, ILWL336, ILWL339, ILWL401, ILWL412, ILWL419, ILWL442 | *L. ervoides*,  *L. culinaris* ssp. *tomentosus*,  *L. nigricans* |
| L | ILWL14, ILWL15, ILWL191, ILWL431, ILWL428, ILWL429, ILWL18, ILWL53, | *L. lamottei*,  *L. nigricans*,  *L. ervoides* |
| M | ILL10829, PRECOZ | *L. culinaris* ssp. *culinaris* |
| N | ILWL247, ILWL86, ILWL121, ILWL67, EC718422, EC718427, EC718429, EC718432, ILWL95, ILWL117, ILWL385, ILWL443, ILWL384, ILWL402, ILWL416, ILWL469, ILWL481, ILWL487, ILWL490, ILWL486, ILWL488, ILWL343, ILWL337, ILWL338, EC718435, ILWL16, ILWL13, ILWL20, ILWL15, ILWL59, ILWL60, ILWL55, ILWL55(2), ILWL56, ILWL65 | *L. culinaris* ssp. *orientalis*,  *L. culinaris* ssp. *tomentosus*,  *L. ervoides*,  *L. nigricans*,  *L. culinaris* ssp. *odemensis* |
