# Supplementary material for: Global Wild Annual Lens Collection: A Potential Resource for Lentil Genetic Base Broadening and Yield Enhancement
Source: PLoS One. 2014 Sep 25;9(9):e107781. doi: 10.1371/journal.pone.0107781 (PMC4177869; doi:10.1371/journal.pone.0107781)
Supplement: Table S4 — Clustering of entire Lens collection based on quantitative agro-morphological data. (DOCX) [file pone.0107781.s004.docx]

**Table S4: Clustering of entire *Lens* collection based on quantitative agro-morphological data.**

| **Major cluster** | **Cluster** | **Sub**  **cluster** | **groups** | **Out**  **groups** | **clutch** | **Sub**  **clutch** | **Genotypes** | **Species** |
| --- | --- | --- | --- | --- | --- | --- | --- | --- |
| I |  |  |  |  |  |  | ILWL418 (367), ILWL292(340) | *L. ervoides* |
| II | A |  |  |  |  |  | ILWL401 (358), ILWL321 (343), ILWL58 (295), ILWL269 (333), ILWL51 (287) | *L. ervoides* |
|  | B | B1 | a | a1 |  |  | EC718428 (381), ILWL384 (144) | *L. ervoides*, *L. culinaris* ssp. *orientalis* |
|  |  |  |  | a2 |  | i | ILWL135 (312), ILWL308 (234), ILWL251 (328), ILWL406 (359) | *L. ervoides*, *L. culinaris* ssp. *tomentosus*, |
|  |  |  |  |  |  | ii | ILWL397 (354), EC718694 (217), ILWL260 (329), ILWL400 (357), ILWL246 (82), EC718430 (383), ILWL155 (318), ILWL165 (188), ILWL50 (286), EC718692 (405), ILWL407 (360), ILWL334 (345), ILWL414 (365), ILWL270 (334), ILWL83 (183) | *L. ervoides*, *L. culinaris* ssp. *odemensis*, L*. culinaris* ssp. *orientalis*,  *L. lamottei* |
|  |  |  | b | b1 |  |  | ILWL388 (351), ILWL490 (174), ILWL378 (140), ILWL372 (135), ILL8006 (3), ILWL286 (92) | *L. ervoides*, *L. culinaris* ssp. *orientalis*, *L. culinaris* ssp. *culinaris* |
|  |  |  |  | b2 | b2a | iii | ILWL370 (133), ILWL366 (130), ILWL255 (87) | *L. culinaris* ssp. *orientalis* |
|  |  |  |  |  |  | iv | EC718439 (391), ILWL294 (341), ILWL476 (164), L830 (1) | *L. ervoides*, *L. culinaris* ssp. *orientalis*, *L. culinaris* ssp. *culinaris* |
|  |  |  |  |  | b2b | v | ILWL44 (282), ILWL37 (257), ILWL410 (362), ILWL29 (399), ILWL138 (314), ILWL323 (344), EC718446 (218), EC718309 (212), ILWL470 (209), ILWL357 (200), ILWL249 (85), ILWL411 (363), ILWL127(306), ILWL480 (235), ILWL230 (76), ILWL141 (316), ILWL134 (311), ILWL460 (262), EC718440 (392), ILWL126 (305), ILWL10 (176), ILWL64(299), ILWL40 (278), ILWL159 (321), ILWL67 (301), ILWL265 (332), ILWL247(83), ILWL203 (191), EC718617 (175), ILWL350 (121), ILWL346 (117) | *L. ervoides*, *L. nigricans*, *L. lamottei* , *L. culinaris* ssp. *tomentosus*, *L. culinaris* ssp. *odemensis*, *L. culinaris* ssp. *orientalis* |
|  |  |  |  |  |  | vi | ILWL395 (352), ILWL90 (219), ILWL133 (310), ILWL130 (308), ILWL340 (350), ILWL234 (327), EC718429 (382), ILWL121 (223), ILWL191 (260), ILWL111 (259), ILWL28 (252), EC718272 (270), ILWL468 (208), ILWL166 (189), ILWL237 (195), ILWL47 (284), EC718693 (216), ILWL54 (290), ILWL42 (280), ILWL156 (319), ILWL128 (307), ILWL162 (322), ILWL41 (279), ILWL261 (330), EC718273 (271), ILWL398 (355) | *L. ervoides*, *L. culinaris* ssp. *tomentosus*, *L. nigricans*, *L. culinaris* ssp. *odemensis* |
|  |  | B2 | c |  |  |  | EC718449 (396) | *L. ervoides* |
|  |  |  | d | d1 | d1a | vii | ILWL330 (108), ILWL157B (60), ILWL317(103), ILWL374 (137) | *L. culinaris* ssp. *orientalis* |
|  |  |  |  |  |  | viii | ILWL324 (104), ILWL302 (98), ILWL290 (93), ILWL442 (371), ILWL469 (162), ILWL326 (106), ILWL344 (115), ILWL301 (97), ILWL355(124), ILWL332 (110), ILWL467 (161), ILWL417 (149) | *L. culinaris* ssp. *orientalis*, *L. ervoides* |
|  |  |  |  |  | d1b | ix | ILWL475 (163), ILWL7 (8), ILWL443 (155), ILWL147(55), ILWL313 (102), ILWL310 (100), ILWL157A (59), ILWL408 (361), ILWL146 (54), ILWL345 (116), ILWL412 (364), ILWL152 (58), ILWL312 (101), ILWL277 (89), ILWL424 (152), ILWL423 (151), ILWL425 (153), ILWL488 (172), PRECOZ (4), ILL10829 (2) | *L. culinaris* ssp. *orientalis*, *L. ervoides*, *L. culinaris* ssp. *culinaris* |
|  |  |  |  |  |  | x | ILWL422(150), ILWL353 (122), ILWL354 (123), ILWL341 (112), ILWL309 (99), ILWL297 (96), ILWL402 (147), ILWL329 (107), ILWL481 (167), ILWL349 (120), ILWL416 (148), ILWL342 (113), ILWL335 (111), ILWL295 (95), ILWL293 (94), ILWL456 (159), ILWL178 (64), ILWL148 (56), ILWL150 (57), ILWL337 (347), ILWL143 (52), ILWL176 (62), ILWL281 (91), ILWL278 (90), ILWL426 (154), ILWL487 (171), ILWL486 (170), ILWL419 (368), ILWL338 (348) | *L. culinaris* ssp. *orientalis*, *L. ervoides* |
|  |  |  |  | d2 | d2a | xi | ILWL122 (49), ILWL113 (46), ILWL93 (33), ILWL103 (41), ILWL87 (29), ILWL08 (239), ILWL81 (182) | *L. culinaris* ssp. *orientalis*, *L. nigricans*, *L. culinaris* ssp. *odemensis* |
|  |  |  |  |  |  | xii | EC718436 (388), ILWL120 (222), ILWL204 (325), ILWL431 (403), ILWL315 (261), EC718271 (269), ILWL78 (21), ILWL382 (143), EC718424 (377), ILWL23 (248), ILWL199 (230), ILWL343 (114), ILWL80 (23), ILWL75 (18), EC718435 (387), ILWL276 (337), ILWL439(369), ILWL444 (156), ILWL108 (185), ILWL27 (273), ILWL16 (241), ILWL28 (274), ILWL97 (221), ILWL17(244), ILWL92 (303), EC718441 (393), EC718438 (390), EC718437 (389), EC718264 (264), ILWL222 (193), ILWL478 (166), ILWL455 (158), ILWL457 (373), ILWL428 (400), EC718434 (386), ILWL474 (263), ILWL79 (22), ILWL4 (6), ILWL6 (7), EC718423 (376), EC718422 (375) | *L. ervoides*, *L. culinaris* ssp. *tomentosus*, *L. lamottei*, *L. nigricans*, *L. culinaris* ssp. *orientalis*, *L. culinaris* ssp. *odemensis* |
|  |  |  |  |  |  | xiii | ILWL18 (245), ILWL77 (20), ILWL192 (69), ILWL125 (51), ILWL242 (79), ILWL182 (67), ILWL63 (298), ILWL385 (145), ILWL244 (81), ILWL104 (42), ILWL76 (19), ILWL180 (65), ILWL65 (300), ILWL30 (276), ILWL39 (181), ILWL29 (275), ILWL94 (34) | *L. nigricans*, *L. culinaris* ssp. *orientalis*, *L. ervoides*, *L. culinaris* ssp. *odemensis* |
|  |  |  |  |  |  | xiv | ILWL98 (38), ILWL95 (35), ILWL88 (30), WL101(39), ILWL438 (205), ILWL109 (45), ILWL436 (204), ILWL183 (68), ILWL115 (47), ILWL145 (53), ILWL62 (11), ILWL91 (302), ILWL06 (238), ILWL09 (240) | *L. culinaris* ssp. *orientalis*, *L. culinaris* ssp. *odemensis*, *L. ervoides*, *L. nigricans* |
|  |  |  |  |  |  | xv | ILWL35 (180), ILWL3 (5), ILWL377 (139), ILWL24 (249), EC718432 (384), ILWL43 (281), ILWL462 (206), EC718267 (267), EC718310 (213), ILWL241 (78), ILWL430 (402), ILWL185 (324), EC718673 (237), ILWL194 (225), ILWL254 (197), ILWL373 (136), EC718266 (266), EC718442 (394), ILWL22 (247), ILWL450 (372), ILWL472 (210), ILWL53 (289), ILWL477 (165), EC718275 (272), ILWL441 (370), ILWL331 (109), ILWL437 (404), ILWL142 (317), ILWL280 (338), ILWL25 (250), ILWL314 (198), ILWL336 (346) | *L. culinaris* ssp. *odemensis*, *L. culinaris* ssp. *orientalis*, *L. nigricans*, *L. ervoides*, *L. lamottei*, *L. culinaris* ssp. *tomentosus* |
|  |  |  |  |  |  | xvi | ILWL282 (231), ILWL227 (74), ILWL221 (192), EC718427 (380), ILWL123 (304), ILWL271(335), ILWL46 (283), ILWL116 (186), ILWL253 (86), ILWL86 (28), ILWL365 (129), ILWL27 (251), ILWL380 (141), ILWL57(294), ILWL34 (256), EC718425 (378), ILWL376 (138), ILWL307 (233), ILWL274 (336), ILWL100 (184), ILWL429 (401), ILWL284 (339), ILWL69 (12), ILWL30 (253), ILWL248 (84), ILWL299 (342), ILWL38 (258), ILWL362 (202), ILWL361 (201), ILWL81 (24), ILWL36 (277), ILWL21 (178), ILWL71 (14), ILWL70 (13), ILWL485 (169) | *L. culinaris* ssp. *tomentosus*, *L. culinaris* ssp. *orientalis*, *L. culinaris* ssp. *odemensis*, *L. ervoides*, *L. nigricans*, *L. lamottei* |
|  |  |  |  |  | d2b | xvii | ILWL489 (173), ILWL484 (168), ILWL466 (160) | *L. culinaris* ssp. *orientalis* |
|  |  |  |  |  |  | xviii | ILWL55(2) (292), ILWL55 (291), ILWL59 (296), ILWL14 (397), ILWL371 (134), ILWL96 (36), ILWL367 (131), ILWL117(48), ILWL171 (61), ILWL19 (246) | *L. ervoides*, *L. lamottei*, *L. culinaris* ssp. *orientalis*, *L. nigricans* |
|  |  |  |  |  |  | xix | ILWL15 (398), ILWL60 (297), ILWL56 (293), ILWL20 (177), ILWL15 (243), ILWL13 (242), ILWL8 (9) | *L. lamottei*, *L. ervoides*, *L. culinaris* ssp. *odemensis*, *L. nigricans*, *L. culinaris* ssp. *orientalis* |
|  |  |  |  |  |  | xx | ILWL181 (66), ILWL105 (43), ILWL219 (71), ILWL82 (25), ILWL106 (44), ILWL72 (15), ILWL447 (157), ILWL196 (227), ILWL97 (37), ILWL235 (194), ILWL386 (146), ILWL92 (32), ILWL73 (16), ILWL348 (119), ILWL225 (73), ILWL85 (27), ILWL347 (118), ILWL220 (72), ILWL124 (50), ILWL84 (26), ILWL74 (17), ILWL360(127), ILWL177 (63), ILWL55 (10) | *L. culinaris* ssp. *orientalis*, *L. culinaris* ssp. *tomentosus*, *L. culinaris* ssp. *odemensis* |
|  |  |  |  |  |  | xxi | ILWL359 (126), ILWL325 (105), ILWL358 (125), ILWL102 (40), ILWL89 (31) | *L. culinaris* ssp. *orientalis* |
|  |  |  |  |  |  | xxii | ILWL305 (232), ILWL149 (224), ILWL195 (226), EC718448 (395), ILWL461 (374), ILWL52 (288), ILWL31 (254), EC718311 (214), EC718308 (211), ILWL381 (142), ILWL409 (203), ILWL415 (366), ILWL232 (326), EC718433 (385), ILWL320 (199), ILWL396 (353), EC718312 (215), ILWL228 (75), ILWL49 (285), ILWL160 (187), ILWL399 (356), ILWL33 (255), EC718426 (379), EC718265 (265) | *L. culinaris* ssp. *tomentosus*, *L. ervoides*, *L. nigricans* , *L. culinaris* ssp. *odemensis*, *L. culinaris* ssp. *orientalis* |
|  |  |  |  |  |  | xxiii | ILWL263 (331), ILWL139 (315), ILWL93 (220), ILWL137 (313), ILWL339 (349), ILWL158 (320), ILWL131 (309), ILWL167 (190), ILWL243 (80), ILWL184 (323), ILWL198 (229), ILWL197 (228), EC718672 (236), ILWL238 (196), ILWL231 (77), ILWL364 (128), ILWL256 (88), ILWL464 (207), ILWL369 (132), ILWL23 (179), ILWL201 (70), EC718270 (268) | *L. ervoides*, *L. culinaris* ssp. *tomentosus* , *L. culinaris* ssp. *odemensis*, *L. culinaris* ssp. *orientalis*, *L. nigricans* |
